# Effective and Economical Option of Anesthesia in Retrograde Intrarenal Surgery

**DOI:** 10.4314/ejhs.v33i6.15

**Published:** 2023-11

**Authors:** Turkan Sadi, Ozan Ekmekcioglu, Ebru Efe Ekmekcioglu, Hakan Ayvaz, Lokman Irkilata, Akkan Avci

**Affiliations:** 1 Kastamonu Anadolu Hospital, Department of Urology, Kastamonu, Turkey; 2 Mersin City Research and Training Hospital, Department of Urology, Mersin, Turkey; 3 Mersin City Research and Training Hospital, Department of Anesthesiology and Reanimation, Mersin, Turkey; 4 Medicana Sivas Hospital, Clinic of Anesthesiology and Reanimation, Sivas, Turkey; 5 Samsun Research and Training Hospital, Department of Urology, Samsun, Turkey; 6 Health Science University, Adana City Research and Training Hospital, Department of Emergency Medicine, Adana, Turkey

**Keywords:** Spinal anesthesia, general anesthesia, urological surgical procedures, cost effectiveness

## Abstract

**Background:**

There is only limited data in the literature showing the effect of anesthesia methods on the success of retrograd intrarenal surgery. The aim of this study was to compare and evaluate retrograd intrarenal surgery cases performed under spinal and general anesthesia in terms of effectiveness, cost, hospitalization time and complications.

**Methods:**

A total of 337 patients who underwent retrograd intrarenal surgery due to kidney stones between 2014 and 2019 were retrospectively evaluated. In our study, the patients were divided into two groups according to the anesthesia method administered: Group 1 consisted of 172 patients who received spinal anesthesia and Group 2 comprised 165 patients administered general anesthesia. Both groups were compared in terms of demographic data, localization and size of stone, radiographic stone density, operation time, complications, need for postoperative analgesia, length of hospitalization, and stone free rate.

**Results:**

The cost of general anesthesia was significantly higher compared to that of spinal anesthesia (p < 0.001). The analgesia application administered within the first six postoperative hours was significantly higher in the general anesthesia group (p < 0.001). In other findings, there was no statistically significant difference between the two groups.

**Conclusion:**

Retrograd intrarenal surgery can be performed with similar safety and effectiveness under both general and spinal anesthesia. However, spinal anesthesia seems to be more advantageous due to the patients' lower need for analgesics in the early postoperative period and the lower cost of the anesthetics used.

## Introduction

In parallel with the advances in technology, the use of minimally invasive methods in the treatment of urinary system tract stone disease is on a continual rise. Today, such minimally invasive techniques as shock wave lithotripsy (SWL), percutaneous nephrolithotomy (PCNL), ureterorenoscopy (URS), retrograde intrarenal surgery (RIRS), and laparoscopic surgery are frequently performed for the treatment purposes (1).

Along with the technical developments in the field of RIRS, there have also been changes in the anesthesia applications and the techniques used (2). Despite being performed by most urologists in daily clinical practice preferably under general anesthesia, RIRS can also be undertaken using spinal or epidural anesthesia, or a combination of both (2,3). However, there is only limited data in the literature showing the effect of anesthesia methods on the success of RIRS. Therefore, this retrospective study evaluates RIRS performed under spinal or general anesthesia in terms of cost, length of hospitalization, and complications.

## Methods

A total of 337 patients who underwent RIRS due to kidney stones between 2014 and 2019 were retrospectively evaluated. Of the patients evaluated according to the American Society of Anesthesiologists (ASA) scoring system, those with an ASA of 4 or above were excluded from the study. Besides, patients with severe hepatic or renal failure, drug allergy, use of antiepileptic drugs, prolonged use of nonsteroidal steroidal anti-inflammatory drugs and opioids, skeletal and spinal deformities, urinary system anomalies (ectopic kidney, renal malrotation, bifid pelvis, duplicated ureter, calyceal diverticula, etc.), morbid obesity, diabetes mellitus, and other neuropathic diseases were excluded. Furthermore, patients who were initially applied spinal anesthesia, but were then sedated or given general anesthesia due to insufficient anesthesia were also excluded. The anesthesia method to be used was determined by the evaluation of the anesthesiologist, and the urologists did not have any effect on this decision. The records of all patients were examined in detail, and their routine preoperative urinary culture, blood biochemistry, complete blood count, urine analysis, and hemostatic parameters were individually evaluated for the exclusion criteria.

Kidney-ureter-bladder (KUB) X-ray, urinary ultrasonography, and non-contrast computed tomography were used for the preoperative radiological evaluation. The stone size was determined by measuring the longest axis of the stone on the radiologically acquired image. The stone localization was classified as lower calyx, middle calyx, upper calyx, renal pelvis, and multiple localizations.

In our study, the patients were divided into two groups according to the anesthesia method administered: Group 1 consisted of 172 patients who received spinal anesthesia, and Group 2 comprised 165 patients administered general anesthesia. Both groups were compared in terms of demographic data, localization and size of the stone, radiographic stone density (Hounsfield unit, HU), operation time, complications, need for postoperative analgesia, length of hospitalization, and stone-free rate. Additionally, the cost of the two groups was compared based on the current prices of the agents administered according to the anesthesia type.

The operation time was recorded from the access of the semi-rigid ureterorenoscopy into the urethra to the insertion of the transurethral catheter. Intraoperative and postoperative complications were evaluated using the Modified Clavien classification. The presence of postoperative fever (body temperature above 38 °C) was noted. All patients were evaluated by KUB X-ray, urinary ultrasonography, and non-contrast CT, if necessary, on the postoperative first day and in the fourth week. Residual stones of >4 mm were also recorded.

The patients were informed in detail about the anesthesia technique that would be administered. The anesthesia procedures were performed by the same anesthesia team without any premedication. All patients were taken to the operating room after administering 7 mL/Kg crystalloid and 10 ml/Kg crystalloid solution during the surgical procedure. Standard electrocardiography (ECG), peripheral oxygen saturation, and blood pressure were measured at five-minute intervals and recorded.

The patients in the spinal anesthesia group were placed in a sitting position, the L3-4 vertebral space was determined, and the subarachnoid space was accessed from the midline using a 25-G spinal needle. After observing free cerebrospinal fluid for about 30 seconds, a total of 3mL/15mg heavy bupivacaine (Heavy Marcaine®; AstraZeneca, Plankstadt, Germany) was injected into the subarachnoid space. The patients were then placed in a supine position, and the anesthesia block was evaluated at the dermatomal level using the “pin-prick” test (analgesia control by needle tip). The level necessary for surgical anesthesia was accepted as T8. The patients with failed spinal anesthesia were excluded from the evaluation.

In the general anesthesia group, induction of anesthesia was achieved by the intravenous (IV) administration of 2 mg/kg propofol (Diprivan®; Fresenius Kabi, Germany), and muscle relaxation was achieved by the 0.6 mg/kg IV rocuronium (Esmeron®; GlaxoSmithKline, England). Rocuronium was also given for muscle relaxation during the operation when necessary. Following the application of an orotracheal tube, anesthesia was maintained with 6% desflurane (Suprane®, Eczabaşı-Baxter, Istanbul, Turkey), 1 µg/kg fentanyl, 50% N_2_O in oxygen, and 50% O_2_ in air (tidal volume 8 mL/kg, frequency = 10/min, EtCO2 = 35–40 mmHg). The start and finish times, the need for analgesia at six hours and at six to 24 hours, and analgesia applications at these times were recorded in all operations.

All surgical procedures were performed by the same surgery team using a C-arm X-Ray device (Siemens, Munchen, Germany). In all operations, a 9.5 Fr 35 or 45 cm access sheath (Cook®; Bloomington, USA) was used. The patients without the use of access sheath were excluded from the study. The operation was started with a 6-8 Fr semi-rigid ureterorenoscopy (Karl Storz®; Tuttlingen, Germany), and the status of the ureter was examined to determine whether there was any ureteral stone and measure the urethral diameter. A 0.035 guidewire (Boston Scientific; Natick, MA) was advanced to the renal pelvis. The access sheath was then inserted into the ureter via the guidewire. Then, the renal pelvis was accessed with a flexible ureterorenoscopy (F-URS) (Karl Storz; Flex X2, 7.5 Fr, Tuttlingen, Germany). Safety was ensured through the C-arm X-ray during all these stages. The stones were fragmented with the holmium laser (Dornier Medilas 30 w, Germany). The presence of only fragments of <5 mm was accepted as a surgical success. At the end of the operation, a 4.8 Fr double-j stent and a 16-18 Fr urethral Foley catheter were inserted in all patients. The double-j stents were removed after two to three-3 weeks.

Ethical approval for this study was granted by Kastamonu University Ethics Committee (protocol no: 2022-KAEK-20).

**Cost calculation**: The prices of drugs and materials used in anesthesia applied to the patients included in the study were calculated. This calculation was made on the basis of the Health Implementation Communiqué published by the Ministry of Health. Expenses calculated in Turkish Lira were converted into American Dollars at the current exchange rate.

**Statistical analysis**: All data were analyzed using IBM SPSS software v. 23. The normality of the variables was tested with the Kolmogorov-Smirnov method. The comparison of the non-normally distributed data was performed with the Mann-Whitney U test. The categorical data were compared using the chi-square test. The results of analyses were expressed as median (min-max) values for quantitative data, and as frequencies (percentages) for categorical data. The significance level was set at p<0.05. The stone localizations were examined with the chi-square test.

## Results

Data on demographic characteristics of the patient groups are given in Table 1. As a result of the analysis, no statistically significant difference was found between the groups in terms of the mean stone size, operation time, length of hospitalization, and radiological stone density (HU). However, the cost of general anesthesia (18.5 USD) was significantly higher compared to that of spinal anesthesia (4.2 USD) (p < 0.001) (Table 1).

**Table 1:** Comparison of demographic and clinical data according to the anesthesia groups

		General	Spinal	Test statistics	p
		(n = 165)	(n = 172)		
Age		42 (22 - 71)	46 (24 - 73)	U = 16539	0.009
Gender				*= 0.141	0.707
	Female	82 (49.7)	89 (51.7)		
	Male	83 (50.3)	83 (48.3)		
Stone size (mm)		13 (10 - 20)	14 (10 - 20)	U = 15715	0.083
Operation time (min)		50 (30 - 120)	50 (30 - 100)	U = 14689	0.572
Length of hospitalization (days)		1 (1 - 4)	1 (1 - 4)	U = 14094	0.664
Stone HU		775 (768 - 784)	775 (768 - 784)	U = 14171.5	0.983
Cost (USD)		18.5 (18 - 22)	4.2 (3.9 - 4.9)	U = 0.000	**<0.001**

U: Mann Whitney U test, HU: Hounsfield unit

* Chi-square test

The analgesia application administered within the first six postoperative hours was significantly higher in the general anesthesia group (p < 0.001). However, no significant difference was found between the two groups in terms of the analgesic application at six to 24 hours (Table 2). There was also no significant difference between the groups in terms of the stone-free and complication rates (Table 2) or stone localization (Table 3).

**Table 2:** Comparison of other investigated parameters according to the anesthesia groups

	General (n = 165)	Spinal (n = 172)	Test statistics	p
Need for anesthetics within the first six hours				
No	28 (17)	147 (85.5)	=158.275	**<0.001**
Yes	137 (83)	25 (14.5)		
Need for anesthetics from the sixth to 24^th^ hours				
No	6 (3.6)	7 (4.1)	=0.000	1.000
Yes	159 (96.4)	165 (95.9)		
Stone-free status				
No	147 (89.1)	155 (90.1)	=0.017	0.897
Yes	18 (10.9)	17 (9.9)		
Complications				
No	161 (97.6)	169 (98.3)	=0.191	0.662
Yes	4 (2.4)	3 (1.7)		

**Table 3:** Evaluation of stone localizations according to the anesthesia groups

Stone localization	Spinal anesthesia (n = 172)	General anesthesia (n = 165)	Total (n = 337)	p
Lower calyx	38 (22.1)	33 (20)	71 (21.1)	0.941
Middle calyx	37 (21.5)	38 (23)	75 (22.3)	
Upper calyx	29 (16.9)	30 (18.2)	59 (17.5)	
Renal pelvis	41 (23.8)	35 (21.2)	76 (22.6)	
Multiple localizations	27 (15.7)	29 (17.6)	56 (16.6)	

According to the modified Clavien classification, none of the patients in the two groups developed any intraoperative or postoperative complications. Fever was found in three patients from the spinal anesthesia group and four patients from the general anesthesia group. These patients were hospitalized for three days and discharged following appropriate antibiotic therapy. All operated patients were radiologically evaluated within the first month in terms of residual stones. Residual stones were detected in 17 patients from the spinal anesthesia group and 18 patients from the general anesthesia group. However, no statistically significant difference was found between the groups in terms of residual stones.

## Discussion

The Guidelines of the European Association of Urology state that although endoscopic interventions for the upper urinary tract stones can be performed under local or spinal anesthesia, these procedures are mostly performed under general anesthesia, and RIRS, SWL and PCNL are regarded as alternative methods in the treatment of renal pelvis stones < 2 cm (4). It is mainly due to its potential to reach the stone in a natural way and to achieve higher success rates with lower morbidity that RIRS has become a widely used treatment method (1). RIRS is a safe surgical procedure although it is vulnerable to potential complications (5).

Although RIRS is a minimally invasive procedure, general anesthesia that is commonly preferred during this procedure complicates the process, and therefore it has been argued that the selection of anesthesia methods to be used should be considered well (6). Undoubtedly, in the choice of the anesthesia method, the medical features of the patient and the experience and skills of the surgeon and anesthetist are effective.

General anesthesia in RIRS is preferred because it provides easier access to stones by allowing the anesthesiologist to control the tidal volume and diaphragm movements. Zeng et al (2). suggested that undesired traumas might occur due to insufficient anesthesia and variable respiratory movements in the cases of RIRS performed with regional anesthesia and recommended the use of regional anesthesia in patients at high risk of complications associated with general anesthesia. However, Baran et al (7). evaluated 1,467 cases and reported that RIRS could be safely performed under both general and spinal anesthesia. In addition, the authors reported that although surgical success was increased by creating apnea and preventing diaphragm and kidney mobility, the risk of developing pneumothorax, subcutaneous emphysema, hypercapnia, and acidosis should be taken into account (8). In our patient group, no statistically significant difference was found between the spinal and general anesthesia groups. None of the patients who underwent RIRS with spinal anesthesia had any surgical problems related to respiratory movements. In some of our patients in the spinal anesthesia group, we increased the success of the operation by facilitating access to the stone and performing short-term respiratory controls through verbal communication. Supporting our results, Bosio et al (9). found no difference between general and spinal anesthesia in terms of the success of RIRS and complications.

With the developing technology in the medical sector, increased treatment effectiveness has also led to increased costs. Therefore, it is ideal to combine effective treatment with low cost. Although this situation varies between countries, serious differences may present between the financial burden brought by general and spinal anesthesia in RIRS. In a prospective study with patients undergoing RIRS, the mean cost was found as 13.9 USD in the general anesthesia group and 3.5 USD in the spinal anesthesia group (p < 0.001). Similarly, in their prospective study, Zeng et al. (2) reported the mean anesthesia cost as 391.9 ± 59.1 USD and 183.8 ± 31.4 USD in RIRS performed with general anesthesia and combined spinal-epidural anesthesia, respectively (p < 0.001). In another study, the cost burden of general anesthesia and spinal-epidural anesthesia was found to be similar in RIRS (9). In our study, the mean anesthesia cost was significantly lower in the spinal anesthesia group (4.2 USD vs. 18.5 USD; p < 0.001).

In our spinal anesthesia group, the effect of the agent administered was sufficient to provide anesthesia for a duration of 120-150 minutes. We did not experience time problems in terms of completing the surgical procedure in any of the patients in the spinal anesthesia group. In addition, we found that the patients had a significantly lower need for analgesics, especially within the first six hours due to the continuation of the anesthetic effect through gradual reduction which stops immediately in general anesthesia, as expected (p < 0.001). Similarly, Karabulut et al. (6) reported a significantly lower need for analgesics in the spinal anesthesia group within the postoperative first 24 hours (p < 0.05). In a study by Zeng et al. (2), no significant difference was found between the combined spinal-epidural anesthesia and general anesthesia groups within the postoperative 24-hour in terms of the need for analgesics, which was explained by the nature of RIRS. In our study, no significant difference was found between the two groups in terms of the need for anesthesia between the postoperative sixth and 24^th^ hours (p =1.00).

As for the limitations of the study, we did not consider the factors affecting the patients' preference of the anesthesia method related to the operation and did not objectively evaluate the satisfaction of the surgeon or patients. In conclusion, RIRS can be performed with similar safety and effectiveness under both general and spinal anesthesia. However, spinal anesthesia seems to be more advantageous due to the patients' lower need for analgesics in the early postoperative period and the lower cost of the anesthetics used. The studies in the literature on this issue are limited, and therefore our results should be supported by further prospective randomized studies with larger case series.
